# A Novel Immune-Related Gene Signature to Identify the Tumor Microenvironment and Prognose Disease Among Patients With Oral Squamous Cell Carcinoma Patients Using ssGSEA: A Bioinformatics and Biological Validation Study

**DOI:** 10.3389/fimmu.2022.922195

**Published:** 2022-07-06

**Authors:** Yun Chen, Yunzhi Feng, Fei Yan, Yaqiong Zhao, Han Zhao, Yue Guo

**Affiliations:** ^1^Department of Stomatology, The Second Xiangya Hospital, Central South University, Changsha, China; ^2^Hunan Key Laboratory of Oral Health Research, Hunan 3D Printing Engineering Research Center of Oral Care, Hunan Clinical Research Center of Oral Major Diseases and Oral Health, Xiangya Stomatological Hospital, Xiangya School of Stomatology, Central South University, Changsha, China; ^3^Department of Ophthalmology, Eye, Ear, Nose, and Throat Hospital of Fudan University, Shanghai, China; ^4^Laboratory of Myopia, National Health Commission (NHC) Key Laboratory of Myopia (Fudan University), Chinese Academy of Medical Sciences, Shanghai, China; ^5^Shanghai Key Laboratory of Visual Impairment and Restoration, Fudan University, Shanghai, China

**Keywords:** oral squamous cell carcinoma, immune-related gene, immune infiltration, prognostic biomarker, single-sample gene set enrichment analysis

## Abstract

Oral squamous cell carcinoma (OSCC) is the most invasive oral malignancy in adults and is associated with a poor prognosis. Accurate prognostic models are urgently needed, however, knowledge of the probable mechanisms behind OSCC tumorigenesis and prognosis remain limited. The clinical importance of the interplay between the immune system and tumor microenvironment has become increasingly evident. This study explored immune-related alterations at the multi-omics level to extract accurate prognostic markers linked to the immune response and presents a more accurate landscape of the immune genomic map during OSCC. The Cancer Genome Atlas (TCGA) OSCC cohort (n = 329) was used to detect the immune infiltration pattern of OSCC and categorize patients into two immunity groups using single-sample gene set enrichment analysis (ssGSEA) and hierarchical clustering analysis. Multiple strategies, including lasso regression (LASSO), Cox proportional hazards regression, and principal component analysis (PCA) were used to screen clinically significant signatures and identify an incorporated prognosis model with robust discriminative power on the survival status of both the training and testing set. We identified two OSCC subtypes based on immunological characteristics: Immunity-high and immunity low, and verified that the categorization was accurate and repeatable. Immunity_ high cluster with a higher immunological and stromal score. 1047 differential genes (DEGs) integrate with immune genes to obtain 319 immue-related DEGs. A robust model with five signatures for OSCC patient prognosis was established. The GEO cohort (n = 97) were used to validate the risk model’s predictive value. The low-risk group had a better overall survival (OS) than the high-risk group. Significant prognostic potential for OSCC patients was found using ROC analysis and immune checkpoint gene expression was lower in the low-risk group. We also investigated at the therapeutic sensitivity of a number of frequently used chemotherapeutic drugs in patients with various risk factors. The underlying biological behavior of the OSCC cell line was preliminarily validated. This study characterizes a reliable marker of OSCC disease progression and provides a new potential target for immunotherapy against this disease.

## Introduction

Oral squamous cell carcinoma (OSCC) is the most common malignant tumor found in the oral cavity, occurring on the lips, tongue, palate, cheek mucosa, gum, the floor and vestibule of the mouth, and retromolar area (1). The number of new OSCC cases worldwide reached 377,713 in 2020, most of whom were concentrated in South Asia, and resulted in 177,757 deaths (2). OSCC risk factors include smoking, alcohol intake, chewing betel nut, and human papillomavirus (HPV) infection (3, 4). The prognosis of patients with OSCC is usually evaluated based on patient age, tumor histological grade, TNM stage, and both smoking and drinking status (5). While OSCC diagnosis and treatment have made great progress in recent years, OSCC prognosis has not improved significantly (6). Recent clinical data has shown that the morbidity and mortality of OSCC are still high, the 5-year patient survival rate is approximately 50% (7), and even after standard treatment, the recurrence rate is as high as 18–76% (8). Thus, it is critical that an effective OSCC prognostic model is identified that will effectively predict OSCC outcomes and guide patient treatment.

The immune system’s impact on cancer progression has been a research hotspot for more than a century. Immune checkpoint gene inhibitors are an extensive and effective immunotherapy that block the inhibitory immune checkpoint pathway and reactivate the anti-cancer immune response. The anti-PD-1 immune checkpoint inhibitors, nivolumab and pembrolizumab, are effective treatments for recurrent head and neck squamous cell carcinoma (HNSCC) which is nonresponsive to platinum chemotherapy (9). However, the effectiveness of immunotherapy is dependent on the reactivation of the host immune response in the tumor microenvironment (TME) (10, 11). Recent studies show that the TME, which consists of immune cells, cytokines secreted by immune cells, and immune-related pathways, plays an important role in predicting disease outcomes and evaluating the impact of therapy (12). OSCC is a highly immunogenic tumor and its TME is characterized by changes in the immune cell population, immune checkpoints, and tumor or microenvironmental factors that alter the TME balance and promote immunosuppression, allowing tumors to escape from immune surveillance (13, 14). The microenvironment of solid tumors is more complex than malignant hematological tumors and may directly regulate host immune responses (15). Accumulating studies indicate that immune cell infiltration plays a key role in the prognosis of many tumors, including OSCC (13, 16, 17). The ratio of helper T cells (Th17) to regulatory T cells (Treg) is an important factor affecting OSCC prognosis. Immune-related genes are also closely associated with tumor occurrence and development (18, 19). At present, there are few robust prognostic models based on immune-related genes that can be used to prognose patients with OSCC. Thus, it is urgent that new and reliable immune-related prognostic markers are developed to analyze the relationship between immune-related genes and prognosis and to provide clues for characterizing immune infiltration during OSCC.

This study describes the potential use of immune-related gene profiling of OSCC patients from the TCGA database for disease prognosis and diagnosis. The single-sample gene set enrichment analysis (ssGSEA) method was used to classify OSCC patients into two distinct clusters, immunity-high and immunity-low. The molecular and immune patterns of these clusters were validated using the ESTIMATE and CIBERSORT algorithm. Least absolute shrinkage and selection operator (LASSO) regression and Cox regression analysis were used to establish an immune-related gene prognostic model, which was further validated in the GSE41613 dataset. In addition, a nomogram was used to predict the 1-, 3-y, and 5-year overall survival rates of OSCC patients. The immune checkpoint gene profile was compared between the high- and low-risk groups. Finally, mRNA and protein expression of these genes was assessed in four OSCC cell lines and their biological functions was measured using invasion and migration experiments.

## Materials and Methods

### Patients and Datasets

Transcriptome expression data [fragments per kilobase million (FPKM) value] and clinical OSCC information were downloaded from the TCGA database (https://tcga-data.nci.nih.gov/tcga/), and transcriptome expression data and survival information were obtained from the GEO database (https://www.ncbi.nlm.nih.gov/geo/). The data of 331 OSCC samples and 32 normal samples was obtained from TCGA-HNSC cohort. The following were used as inclusion criteria: (1) histologically verified primary OSCC; (2) patients have mRNA expression profiles and corresponding clinical data. Samples with no data on survival status or survival time were excluded from this study. Finally, 329 individuals with OSCC were enlisted for further study, along with clinicopathological data such as age, gender, TNM stage, and grade. We chose GEO datasets that met the following criteria: (1) histologically verified primary OSCC; (2) sample size in the dataset was more than 80; (3) gene expression profiling data; (4) prognostic data from patients. Finally, OSCC samples from GSE41613 with 97 OSCC samples (GPL10558 platform, Illumina HumanHT-12V4.0 expression beadchip) were used as the validation group for this study. The clinical information of the TCGA sets and GEO validation sets was detailed in **Table 1**.The gene expression matrix was then retrieved from the TCGA-OSCC and GSE41613 datasets and created using Strawberry Perl (version 5.32.02). ImmPort (https://www.immport.org/shared/home) datasets were used to compile a list of 1,793 immune-related genes.

**Table 1:** The clnical information of the TCGA sets and GEO validation sets.

Cohort	TCGA, OSCC (n = 329)	GSE41613, OSCC (n = 97)
**Gender** Male	218	68
Female	111	31
**Age (years)**		
<60	135	50
≥60	194	47
**Stage** Stage I-II	72	41
Stage III-IV	257	56

### Clustering of the OSCC Data

The ssGSEA method is a recently proposed algorithm for counting immune cell subsets using RNA samples from various tissue types (including solid tumors) (20). It has less noise and unknown mixture content than other methods, and the cell types are closely related. In this study the ssGSEA method was used to calculate the absolute enrichment fraction of 29 immune cells and their immune-related functions and pathway marker genes in OSCC patients. The R package “GSVA” was used to classify the OSCC samples in the TCGA-OSCC into immunity-high and immunity-low clusters. Principal component analysis (PCA) were used to evaluate each sample in the clusters.

### Evaluating the Efficacy of Immune Clustering

The ESTIMATE algorithm was used to verify the efficacy of ssGSEA clustering. The ESTIMATE, Immune, and Stromal Scores of each OSCC sample in two clusters was determined using R package “ESTIMATE.” Twenty-two types of tumor-infiltrating lymphocytes (TILs) in the two clusters were analyzed using the CIBERSORT algorithm. Expression of the human leukocyte antigen (HLA) family in each cluster was assessed using the R package “ggpubr”.

### Differential Expression Gene Analysis

The R “edgeR” and “limma” packages were used to perform differential expression gene (DEG) analysis, and DEGs (FDR < 0.05 and |log2FC| > 1) were considered significantly changed between the immunity-high and immunity-low clusters. A Venn diagram was used to identify the intersection genes between the DEGs and 1,793 immune-related genes.

### Genomic Alterations and Gene Set Enrichment Analysis

Using the TCGA dataset, we performed copy number variation (CNV) and somatic mutation analysis to determine the correlation between riskscore levels certain genomic OSCC characteristics. The oncoplot was visulized by using R package “maftoos”. The enriched biological process was identified using the Molecular Signatures Database (MSigDB) and the enriched biological process of intersection genes was identified using the R packages “clusterprofiler” and “enrichplot.” Gene enriched biological processes with *P <*0.05 were considered statistically significant.

### Construction of a Clinical Prognostic Signature Based on Immune-Related Gene Expression

The R “Survival” package was used to analyze the clinical data of OSCC samples from TCGA using univariate Cox regression analysis, and immune-related genes that were significantly related to OSCC patient survival were screened out. LASSO regression analysis was used to screen survival-related genes, and the R “glmnet” package was used to identify genes most related to OS using univariate Cox regression analysis. To prevent over-fitting to the maximum extent, 1,000 rounds of cross-validation were used to select the penalty parameters. Based on prognosis-related immune gene expression and coefficients obtained using multivariate Cox regression analysis, an OSCC prognostic marker was constructed using the following formula:


$$\text{Riskscore}\;\left(\text{patients}\right)=\sum_{i=1}^{n}Expression_{Genei}\;\times \;Coefficient_{Genei}\text{ where }“\text{n}”\text{ represents the number of prognostic genes and }“\text{i}”\text{ represents the serial number of each gene}$$

The PCA were used to evaluate each prognostic gene in the clusters. The median riskscore was defined using the R “Survminer” package, and OSCC patients were divided into high- and low-risk groups. The clinical prognostic ability of the riskscore was evaluated using the R “timeROC,” “Survival,” and “Survminer” packages to create time-dependent receiver-operating characteristic (ROC) and Kaplan-Meier (K-M) curves. Using the R “Survival” package, univariate and multivariate Cox regression analyses were performed to evaluate whether key clinical factors such as gender, age and metastatic status could be used as independent predictors of overall OSCC patient survival.

### Construction and Verification of the Nomogram

Nomograms are an effective method to predict OSCC patient survival rates. According to the riskscore, age, sex, primary tumor location and metastatic status, the R “root mean square” and “survival” packages were used to establish a nomogram based on immune-related gene prognostic markers. The calibration curve was used to evaluate the predictive accuracy of the nomogram to distinguish between different patient groups.

### Protein-Protein Network Interactions (PPI)

PPI are recognized and predicted in the search tool for retrieval of interacting genes/proteins (STRING) database (https://string-db.org/). After building the PPI, Cytoscape was used to visualize the PPI and the network’s key genes were identified.

### Exploration of the Model in the Chemotherapy Response

The R package “pRRophetic” was used to computed the the half-maximal inhibitory concentration (IC50) of commonly used chemotherapeutic drugs (21). The IC50 value represents a substance’s ability to block particular biological or metabolic activities. Wilcoxon signed-rank test was used to determine the difference between groups.

### Cell Culture and Transfection

The OSCC cell line, SCC15, and the normal Human Oral Keratinocytes (HOK) cell line were purchased from the Institute of Antibody Engineering, Southern Medical University (Guangzhou, China). The use of all cell lines was approved by the Nanfang Hospital ethics committee. HOK cells were cultivated in DMEM (Gibco, Cat#11995500TB) and SCC15 cells were cultivated in DMEM/F12 (Gibco, Cat#C11330500BT) with 10% fetal bovine serum (FBS) (Gibco, Cat#10099141C), along with 100 U/mL penicillin and streptomycin (Gibco, Carlsbad, CA, USA). The expression vectors for CTSG and TNFRSF4 were designed and synthesized (RiboBio, Guangzhou, China). Over expression and control vectors were transfected into SCC15 cells using the lipofectamine 8000 protocol (beyotime, Cat# C0533). Total RNA and protein were extracted after 24–48 hours.

### RNA Extraction and Quantitative Real-Time PCR (qRT-PCR)

Total RNA extractions were performed using the SteadyPure Quick RNA Extraction kit according to the manufacturer’s instructions (Accurate Biotechnology, Changsha, Hunan, China) and the RNA was reverse transcribed using an Evo M-MLV Mix Kit with gDNA Clean for qPCR (Accurate Biotechnology, Changsha, Hunan, China, AG11728). Amplification and detection were then carried out using the SYBR Green qPCR Kit (Accurate Biotechnology, Changsha, Hunan, China, AG11701). Gene expression was measured and normalized relative to the level of β-actin using the 2^−ΔΔCT^ method after normalization with a reference control. The primer sequences were as follows:

CTSG Forward primer (5′-3′): GAGTCAGACGGAATCGAAACG,
CTSG Reverse primer (5′-3′): CGGAGTGTATCTGTTCCCCTC;
TNFRSF4 Forward primer (5′-3′): GACAGCTACAAGCCTGGAGTTGAC,
TNFRSF4 Reverse primer (5′-3′): ACAGATTGCGTCCGAGCTATTGC;
β-actin Forward primer (5′-3′): GAAGATCAAGATCATTGCTCCT,
β-actin Forward primer (5′-3′): TACTCCTGCTTGCTGATCCA.

### Western Blotting

Total protein was lysed in RIPA lysis buffer (Thermo Scientific, Rockford, IL, USA) containing protease and phosphatase inhibitors. Western blot was then performed as described previously (22). Primary antibodies were used at the manufacturer’s suggested concentrations: CTSG (1:500; 23840-1-AP; Proteintech), TNFRSF4 (1:500; 20006-1-AP; Proteintech) or β-actin (1:5000; Proteintech). Immunoreactive bands were detected using an antirabbit peroxidase-conjugated secondary antibody (1:5000; Proteintech) and visualized using enhanced chemiluminescence (Amersham Imager 600; General Electric Company). Protein band densitometry was performed using Image J.

### Clonogenic Assay

The control group and cells with high CTSG and TNFRSF4 expression were digested with trypsin, and complete medium was used to suspend the cells, adjusting the density to 500 cells/well. The cells were inoculated in a 6-well plate and the medium was changed every 3–4 days. After 1–2 weeks of culture, the cells were removed, washed three times with PBS, fixed in 4% paraformaldehyde for 30 min, stained with 0.1% crystal violet for 30 min, and counted with a microscope and Image J software.

### Transwell Assay

To explore the function of CTSG and TNFRSF4 in an OSCC cell line, the pEXP-RB-Mam-EGFP system was used to overexpress CTSG and TNFRSF4 in SCC15 cells, over expression and control vectors were transfected into SCC15 cells using the lipofectamine 8000 protocol. 24 hours after transfection, the transformed cells (5x10^4^) were suspended in 200 μL DMEM/F12 and inoculated into the upper chamber. For the cell invasion assay, cells were seeded in the upper chambers that were pre-coated with Matrigel (356234, Corning) at a 1:8 dilution and 600 μL complete medium containing 10% was added to the lower chamber. The transwell device was then incubated for 2 days, cells in the inferior chamber were fixed with 4% formaldehyde for 30 min, and dyed with 0.1% crystal violet for 30 min. Cells in the upper chamber were removed with a cotton swab. Cell migration was observed using an inverted microscope (Zeiss, Germany).

### Wound Healing Assay

A horizontal line was drawn behind the 6-well plate and cells were inoculated into the hole. The cells were covered with the hole plate overnight and the following day, the black line behind the vertical orifice plate was scratched with a 100uL tip head so that the scratch intersected with the mark line. After marking, the cells were washed 2–3 times with PBS, the scratched cells were removed, and fresh serum-free medium was added. The cells were incubated for 24 hours, observed under a microscope and photographed. The proportion of migrated cells was calculated using Image J software.

### Statistical Analysis

All statistical analyses were conducted using R software (version 4.0.4). Kaplan-Meier analysis and the log-rank testing were used to assess survival and compare the difference in survival between the clusters and risk groups. Two-tailed *P <*0.05 was considered statistically significant.

## Results

### Construction and Validation of OSCC Clustering

**Figure 1:**
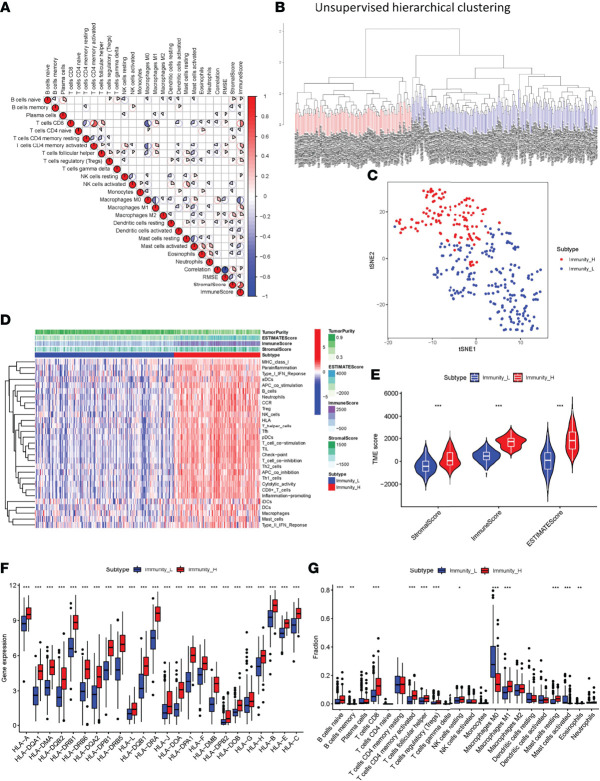
Construction and verification of oral squamous cell carcinoma clustering. **(A)** All 22 invading immune cells are represented by a correlation matrix. Immune cells were shown to be favorably associated and are represented in red, while others were found to be negatively related and are represented in blue. The threshold was set at *P < *0.05. **(B)** Using ssGSEA analysis, gene expression data from OSCC patients were divided into two clusters. **(C)** The PCA plot of the distribution status of the two OSCC clusters. **(D)** The heatmap showed that the 29 immune-related cell types had high expression in the high-immune cell infiltration group (Immunity-high), and low expression in the low immune cell infiltration group (Immunity-low). The tumor purity and ESTIMATE, Immune, and Stromal Scores of each patient are shown with clustering information using the ESTIMATE algorithm. **(E)** The violin plot shows the difference in the ESTIMATE, Immune, and Stromal Scores between the two clusters. **(F)** The box plot shows a statistically significant difference in HLA family expression. **(G)** The box plot shows a statistical difference in immune cell infiltration between the two clusters. *P < 0.05, **P < 0.01, ***P < 0.001.

Samples from 329 OSCC patients were obtained from TCGA database. SsGSEA was used to quantify the OSCC sample RNA-seq data and the infiltration level of 29 immune cell types was obtained. A heatmap was created to depict the differential correlation patterns among the immune cell landscape in the TME (**Figure 1A**). The ssGSEA score of each OSCC sample was calculated and used to divide the samples into immunity-high (n=126) and immunity-low (n=203) clusters with different immune infiltration patterns based on the unsupervised hierarchical clustering algorithm (cutoff=1.0) (**Figures 1B, C**). To verify the feasibility of the clustering results, the ESTIMATE algorithm was used to calculate tumor purity and Stroma, Immune, and ESTIMATE Scores based on the expression of each OSCC sample. The Stroma, Immune, and ESTIMATE Scores of the immunity-high cluster group were higher than those of the immunity-low group, while the tumor purity was lower (**Figure 1D**). The violin plot also showed that the Stroma, Immune, and ESTIMATE Scores were higher in the immunity-high cluster group than in the immunity-low group (*P* < 0.001, **Figure 1E**). Boxplot showed that the expression of most HLA markers was also higher in the immunity-high cluster group than in the immunity-low group (**Figure 1F**), and the CIBERSORT algorithm showed that the proportion of immune cells was higher in the immunity-high cluster group than in the immunity-low group (all *P <*0.001, **Figure 1G**).

### GSEA Enrichment Analysis

**Figure 2:**
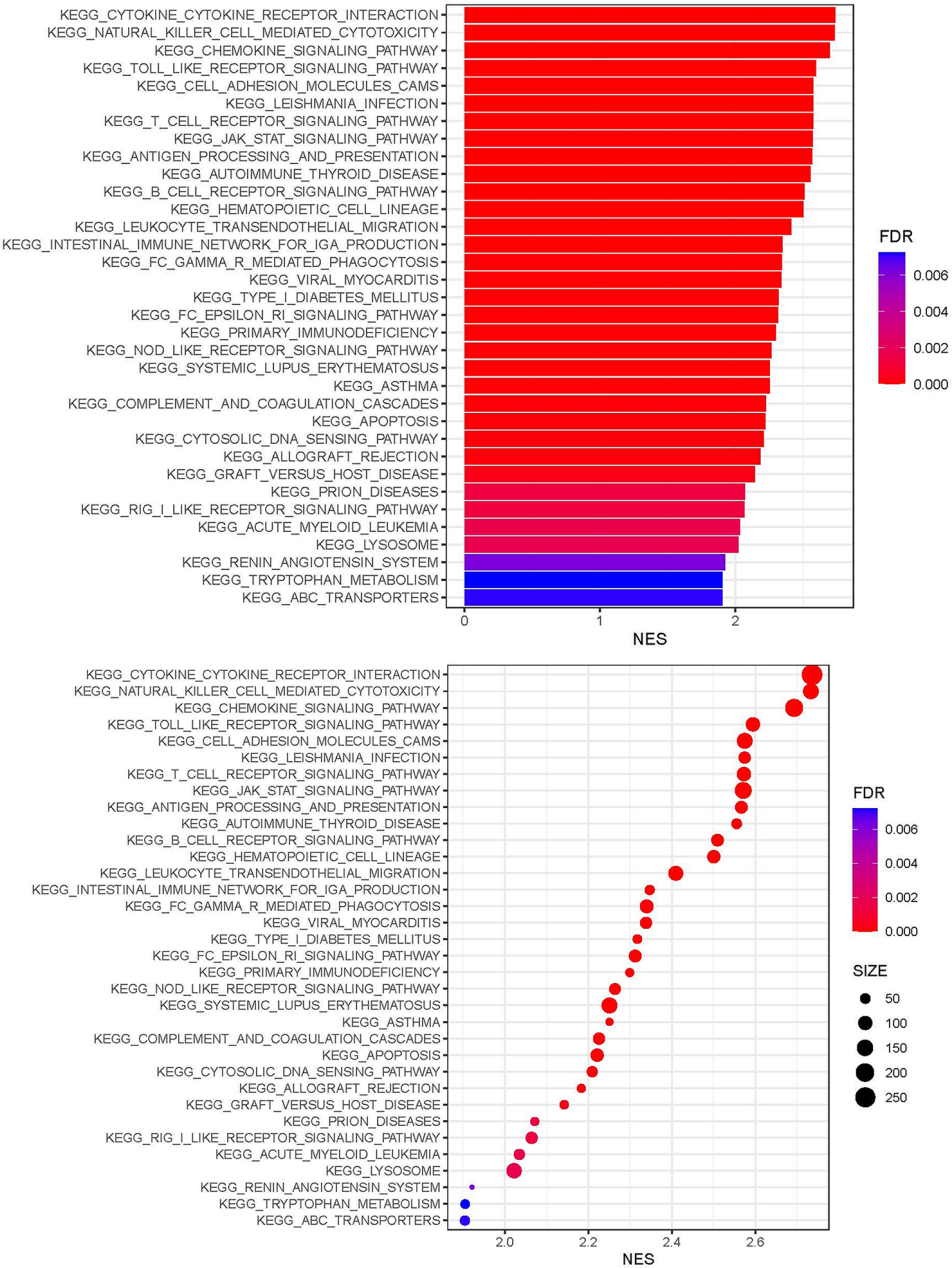
Gene functional enrichment analysis of the immunity-high and immunity-low clusters.

KEGG analysis showed that genes expressed in the immunity-high and immunity-low cluster groups correlated with a number of chemokine, Toll-like receptor, T cell receptor, JAK-STAT, B cell receptor, Fc epsilon RI, NOD-like receptor, and cytosolic DNA sensing signaling pathways (**Figure 2**).

### Identification of Differentially Expressed Immune-Related Genes Between the Immunity-High and Immunity-Low Clusters

**Figure 3:**
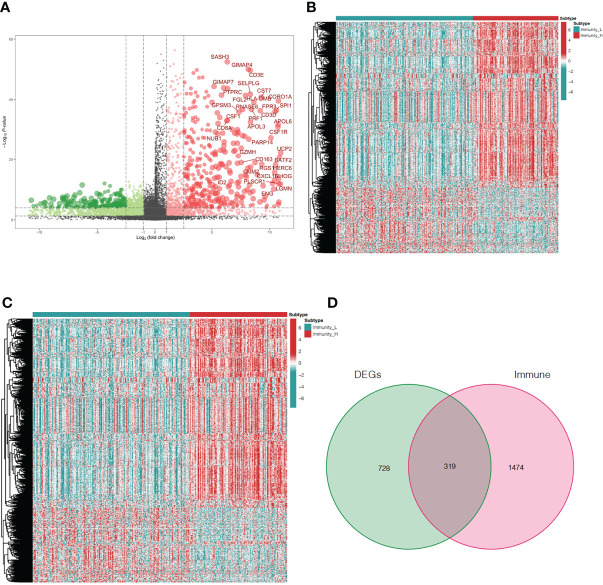
Analysis of differentially expressed immune-related genes in oral squamous cell carcinoma patients. **(A)** The volcano plot shows 761up-regulated genes and 286 down-regulated genes in the immunity-high and immunity-low clusters. Up-regulated and down-regulated genes are represented by red and green bars, respectively. The gene names of genes with |log2FC| > 6 are displayed. **(B)** The heatmap shows the degree of DEG expression in the immune-high and immune-low clusters. **(C)** The heatmap shows immune-related gene expression in the immunity-high and immunity-low clusters. **(D)** The Venn diagram shows 319 genes from both gene sets. DEGs, differentially expressed genes.

To explore differences in DEG expression between the immunity-high and immunity-low clusters in TCGA database, a threshold of FDR < 0.05 and |log2FC| > 1 was used and 1,047 DEGs, including 761 up-regulated and 286 down-regulated genes, were obtained (**Figure 3A**). DEG expression in the immunity-high and immunity-low clusters is shown in **Figure 3B**. In addition, 1,793 immune-related genes were obtained from the ImmPort database. Immune-related gene expression in the immunity-high and immunity-low clusters is shown in **Figure 3C**. A two-way Venn analysis was also conducted using the immune-related genes from the ImmPort database and the DEGs from the immunity-high and immunity-low clusters. This yielded 319 overlapping genes that were defined as true DEGs (**Figure 3D**, **Supplementary Table 1**).

### Screening Immune-Related Gene Prognostic Signature of OSCC

**Figure 4:**
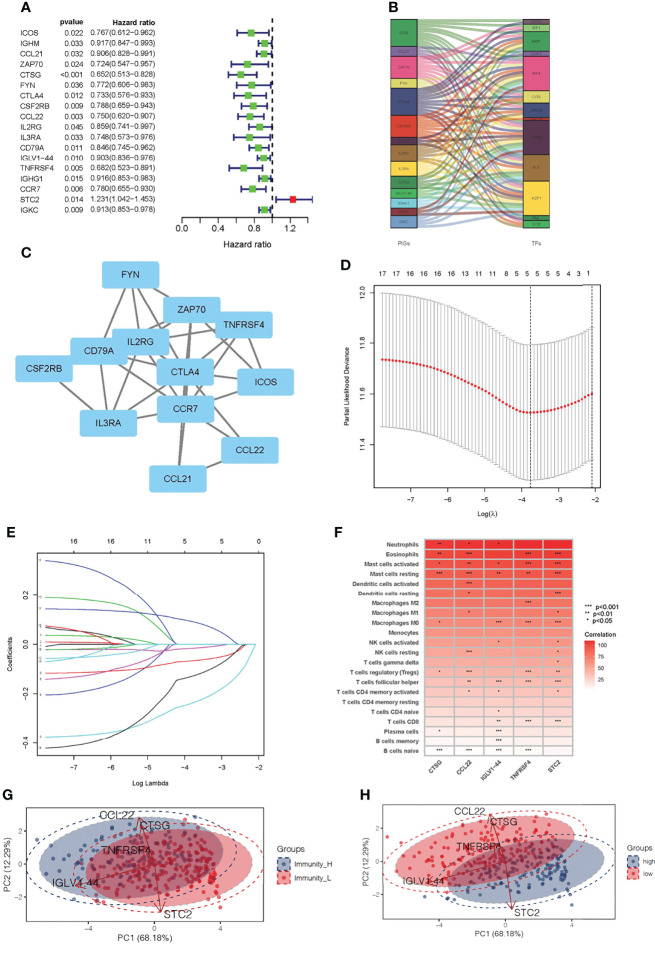
Development of a prognostic signature for oral squamous cell carcinoma based on immune-related genes. **(A)** Using univariable Cox regression analysis, the HR and p-value for the chosen genes in the immune terms. **(B)** The interaction between the 14 immune-related genes and transcription factors is depicted as an alluvial diagram. **(C)** The interaction network of the immune-related prognostic genes. **(D)** The 14 immune-related gene LASSO coefficient profiles. **(E)** 1,000-round cross-validation was used to find the best values for the penalty parameter. **(F)** The heatmap shows the correlation between immune-related prognostic genes and immune cell infiltration. **(G)** PCA results for prognostic genes in two clusters of immunity level in training set. **(H)** PCA results for prognostic genes in high- and low-risk groups in training set.

After integrating clinicopathological information into the gene expression profiles, 329 OSCC patients with complete clinical data were selected for further analysis. Univariate Cox regression analysis was used to detect the roles of 350 overlapping genes to identify immune-related genes that could predict OSCC outcomes. The results indicated that 18 genes were significantly associated with OS (*P <*0.001, **Figure 4A**). An alluvial diagram was then used to show the relationship between the 18 immune-related genes and transcription factors (**Figure 4B**). The interaction network of these genes was established using the STRING database and displayed using Cytoscape (**Figure 4C**). Cross-validation (1,000 rounds) was used to determine the optimal values of LASSO regression algorithms and parameters of the 18 immune-related genes in order to reduce the prognostic signature (**Figures 4D, E**). LASSO regression analysis was then performed and when the five immune-related genes, CTSG, TNFRSF4, IGLV1-44, STC2, and CCL22 were identified, the prognostic model achieved the best performance. The CIBERSORT method was used to assess the correlation between the five prognostic markers and immune cell infiltration. CTSG was associated with resting mast cells and naïve B cells, CCL22 was associated with eosinophils, resting mast cells, activated dendritic cells, resting NK cells, regulatory T cells (Tregs) and naïve B cells, IGLV1-44 was associated with M0 macrophages, follicular helper T cells, plasma cells, and naïve and memory B cells, TNFRSF4 was associated with eosinophils, activated mast cells, M0 and M2 macrophages, Tregs, follicular helper T cells, CD8 T cells, and naive B cells, and STC2 was associated with eosinophils, activated and resting mast cells, resting dendritic cells, follicular helper T cells and CD8 T cells (*P <*0.001, **Figure 4F**). PCA plot was used to validate the distribution of our prognostic genes screened from DEGs in different immunity clusters. This indicates that prognostic genes in the immunity-high and immunity-low group were in two directions in the training cohort (**Figure 4G**). Using the expression of these five genes and their coefficients, the riskscore was calculated for each sample according to the following formula:


$$\begin{array}{lll} \text{Riskscore}=\left(\text{expression of CTSG }*\;-0.235852555481698\right)\;+\;\left(\text{expression of TNFRSF}4\;*\;-0.127188255049261\right)\; & +\;\left(\text{expression of IGLV}1-44\;*\;-0.0157820710202927\right)\;+\;\left(\text{expression of STC}2\;*\;0.0909125160196324\right)\; & +\;\left(expression of CCL22*\;-0.0661762958246814\right) \end{array}$$

This PCA plot indicates that the distribution of our prognostic genes screened from DEGs also have two directions between the low-risk and high-risk group (**Figure 4H**). These findings identified five immune-related genes that are highly sensitive and specific prognostic indicators for OSCC patients.

### Construction and Validation of Prognostic Markers of OSCC Immune-Related Genes

**Figure 5:**
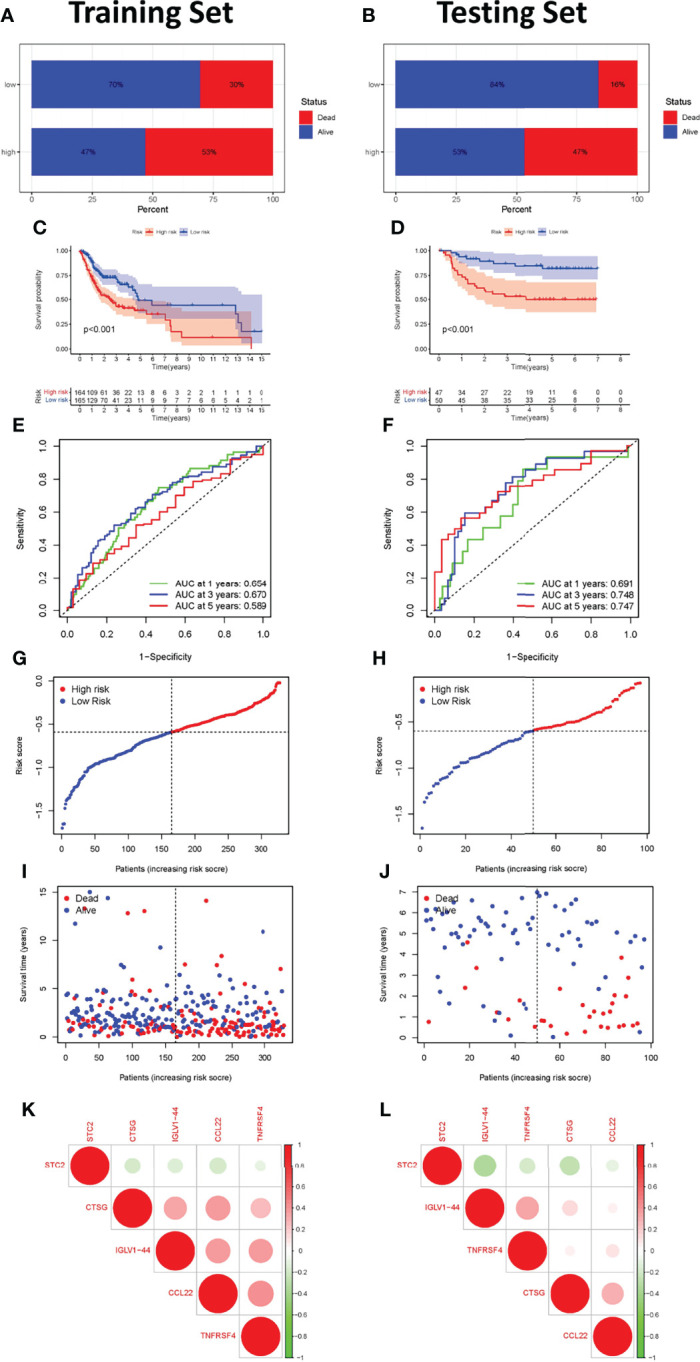
Construction and validation of the immune-related gene prognostic signature in the training and testing sets. The survival status of patients in the high-risk and low-risk groups in the training **(A)** and testing sets **(B)**. Kaplan-Meier survival curves for OSCC patients in the training **(C)** and testing sets **(D)**. The prognostic signature’s time-independent ROC curve at 1-, 3-, and 5-years in the training **(E)** and testing sets **(F)**. Each OSCC sample’s risk curve is reordered by the riskscore in the training **(G)** and testing sets **(H)**. A scatter plot depicts the survival of OSCC samples in the training **(I)** and testing sets **(J)**. Interaction analysis of the immune-related prognostic genes in the training **(K)** and testing sets **(L)**.

The effectiveness and robustness of immune-related genes to predict OS was verified in patients with OSCC. The training set (TCGA cohort) was used to verify the characteristics of immune-related genes and to construct a prognostic model, while the testing set (GSE41613 cohort) was used to independently verify the performance of the prognostic risk model. Using the immune-related prognosis model, the riskscore of each patient in the training set was calculated and the median riskscore was used to divide the patients into a high- and a low-risk group. A higher proportion of patients died in the high- than in the low-risk group (**Figures 5A, B**). The Kaplan-Meier survival curve showed that patients in the high-risk group had markedly poorer OS than those in the low-risk group (*P <*0.001, **Figures 5C, D**). The time-dependent ROC curve was then used to evaluate the accuracy of OS estimates derived from the prognostic model. In the training cohort, the ROC curve showed that the AUC values of riskscore for 1-, 3-, and 5-year survival rates were 0.654, 0.670, and 0.589, respectively (**Figure 5E**). In the testing cohort, the ROC curve showed that AUC values of riskscore for 1-, 3-, and 5-year survival rates were 0.691, 0.748, and 0.747, respectively (**Figure 5F**). The riskscore and survival status of prognostic markers is shown in **Figures 5G–J**, and the correlation between the five-genes prognostic model is shown in **Figures 5K, L**. The riskscore was shown to be a reliable predictor of OS in patients with OSCC.

**Figure 6:**
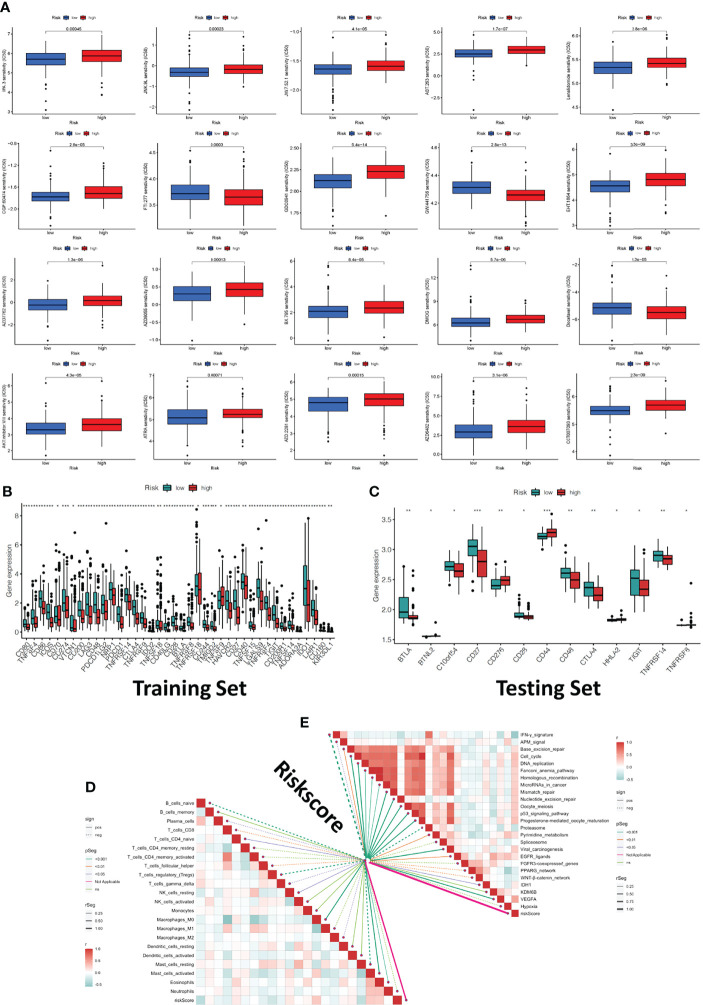
Clinical response to anti-tumor therapy as well as immune checkpoint-related gene expression in the high-risk and low-risk groups. **(A)** The chemotherapy and molecular drugs prediction of risk groups. **(B)** The difference of immune checkpoints expression in risk groups of traing set. **(C)** The difference of immune checkpoints expression in risk groups of testing set. **(D)** Correlations between riskscore and immune infiltration cells. **(E)** Correlations between riskscore and the enrichment scores of immunotherapy-predicted pathways. *P < 0.05, **P < 0.01, ***P < 0.001.

We investigated the association between riskscore and clinical response to chemotherapeutic drugs as well as some immunotherapeutic drugs using the R package “pRRophetic”. By calculating the half-maximal inhibitory concentration (IC50) of anti-tumor drugs, the high-risk and low-risk groups showed a significant difference in sensitivity to 20 chemical or targeted drugs (**Figure 6A**). Most immunological checkpoints were more activated in the low-risk group both in training and testing cohorts. Besides, we also found that the expression of some immune checkpoint gene of immunotherapy, including the rise in CD44, CD276, CD40 and TNFSF9 gene expression in the high-risk group, demonstrated that they had variable effects in each group (**Figures 6B, C**). It meant we could select the most appropriate checkpoint inhibitors for OSCC patients based on their riskscore. According to the CIBERSOFT algorithms, riskscore positively correlated with the infiltration levels of multiple types of immune cell, including the CD4 T cell, B cell, NK cell, dendritic cell, and Macrophage (**Figure 6D**). We also investigated at the links between riskscore and immunotherapy-predicted pathways such oncogenic pathways, targeted therapy-associated gene signatures, and radiation response gene signatures (**Supplementary Table 2**) (23). Riskscore positively correlated with the enrichment scores for almost all anticancer-immunotherapy-related signatures (**Figure 6E**).

### Evaluation of Immune-Related Gene Prognostic Markers as Independent Prognostic Factors for Patients With OSCC

**Figure 7:**
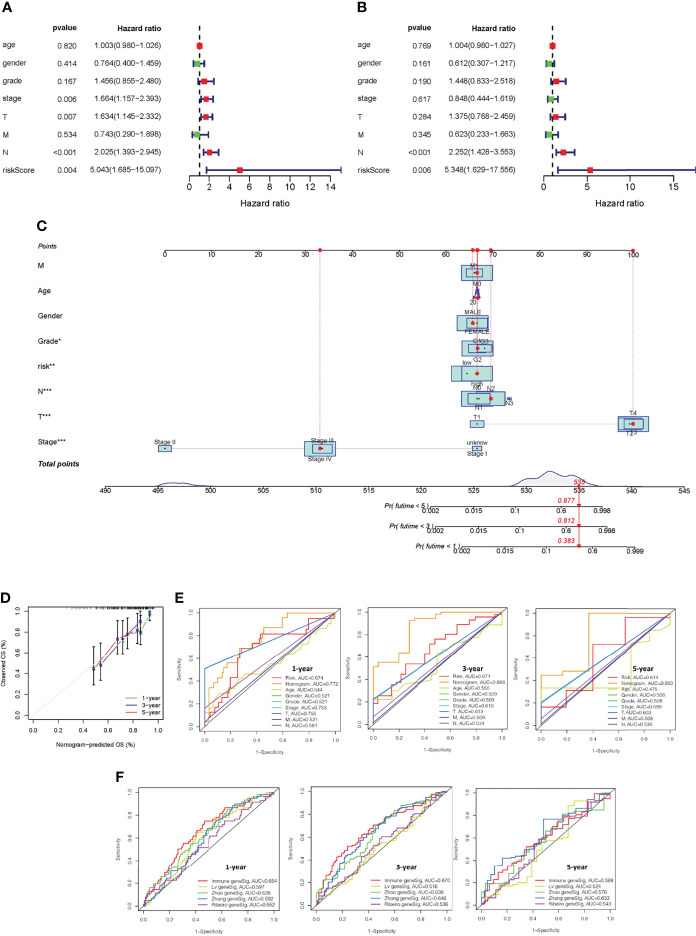
Construction of a nomogram and verification that the immune-related gene prognostic signature is an independent prognostic factor. Univariate **(A)** and multivariate **(B)** Cox regression analysis of the immune-related gene prognostic signature in OSCC patients to determine independent risk variables. **(C)** The development of a nomogram based on the immune-related gene prognostic signature in the TCGA training cohort. **(D)** The calibration curve of the nomogram. **(E)** The combined ROC for riskscore, nomogram, gender, stage, age, and TMN at 1-, 3-, and 5-years. **(F)** Time-independent ROC curves of overall survival for immune-related gene prognostic model, Lv geneSig, Ribeiro geneSig, Zhao geneSig, and Zhang geneSig at 1-, 3-, and 5-years.

Univariate and multivariate Cox regression analysis were used to test whether the five immune-related gene signatures were independent prognostic factors of other features, such as age, gender, and grade. The results indicated that the N stage was an independent prognostic factor (*P <*0.001, **Figures 7A, B**).

### Establishment and Validation of a Nomogram to Predict Overall Survival

To predict the survival of OSCC patients from a clinical perspective, TCGA data was used to construct a nonogram that could estimate the probability of OS lasting 1, 3, and 5 years. Age, gender, stage, TMN status, and riskscore were included as variables to predict prognosis (**Figure 7C**). The 45° line represents the best prediction model, and the resulting calibration plot indicates that the nomogram performed well (**Figure 7D**). We plotted combined ROC for riskscore, nomogram, gender, stage, age, and TMN, and the AUC values of nomogram for predicting the 1-, 3- and 5-year OS rates were 0.772, 0.868 and 0.803, respectively (**Figure 7E**).

The performance of our prognostic model was then compared to four representative prognostic signatures previously generated using the same TCGA-OSCC cohort. Signatures were developed by Lv et al. (24), Ribeiro et al. (25), Zhao et al. (26), and Zhang et al. (27) using eight, seven, four, and five genes, respectively. The AUC of 1-year and 3-year survival of our immune-related gene prognostic model was 0.654 and 0.670, which was significantly higher than the AUC values of the other four prognostic models. The AUC of 5-year survival of our immune-related gene prognostic model was 0.589, which was second only to Zhang geneSig (**Figure 7F**). These findings revealed that our immune-related gene prognostic model is reliable and effective at predicting the prognosis of OSCC patients.

### Prognostic Signature of Immune-Related Genes in Relation to Tumor Mutational Load

**Figure 8:**
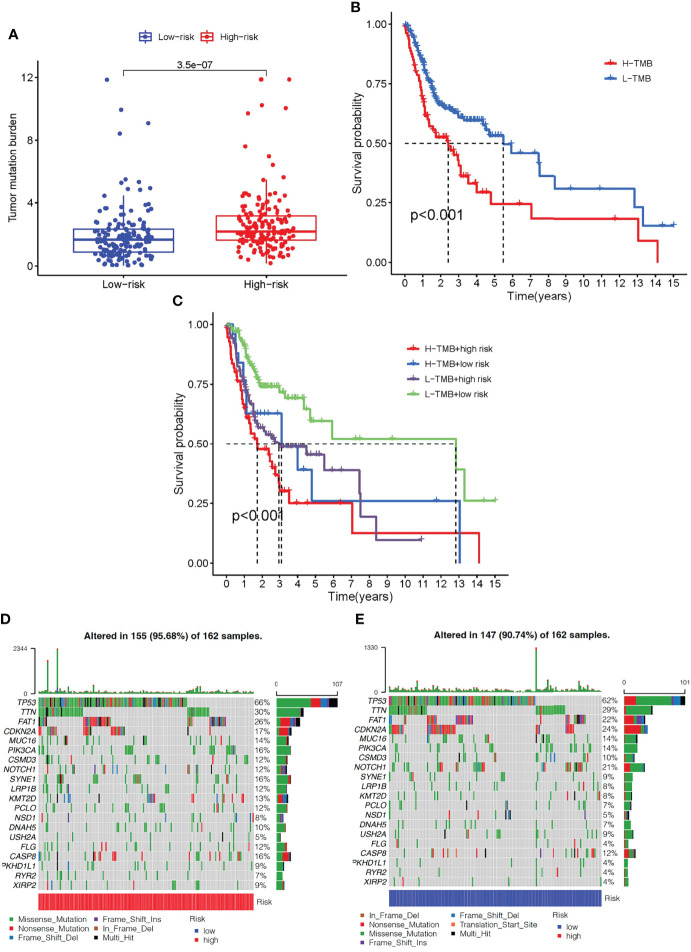
The correlation between the immune-related gene prognostic signature and TMB. **(A)** The box plot for TMB levels among patients in the high- and low-risk groups. **(B)** Kaplan–Meier curves for the high- and low-TMB of OSCC patients. **(C)** Kaplan–Meier curves for OSCC patients by TMB status in the high-risk and low-risk groups. **(D)** The oncoPrint was constructed based on CNV profile in the high-risk scores of OSCC patients. **(E)** The oncoPrint was constructed based on CNV profile in the low-risk scores of OSCC patients. Individual patients are represented in each column. TMB, tumor mutational burden; CNV, copy number variation.

TMB levels in the high- and low-risk groups were measured to determine if there was a correlation between the immune-related gene prognostic signature and the tumor mutational burden (TMB). TMB levels were higher in the high- than in the low-risk group (*P <*0.001, **Figure 8A**). Kaplan-Meier survival analysis also showed that the OS probability was worse in the high- than the low-risk group (*P <*0.001, **Figure 8B**). The predictive profile of immune-related genes were also evaluated in connection with TMB. To investigate the role of TMB status, survival analysis was performed on low-risk group/low-TMB, low-risk group/high-TMB, high-risk group/low-TMB, and high-risk group/low-TMB groups. There was a substantial difference between the four groups (*P <*0.001) (**Figure 8C**). Overall, these findings reveal a link between riskscore and somatic mutation trends. Then, we performed copy number variation (CNV) analysis to determine whether riskscore levels were linked to certain genomic characteristics. Oncogenic driver genes including TP53, TTN, FAT1, PIK3CA, CSMD3, SYNE1, and LRP1B were commonly amplified in high-score samples, whereas CDKN2A, NOTCH1, and USH2A were amplified in low-score samples (**Figures 8D,E**).

### Experimental Validation

**Figure 9:**
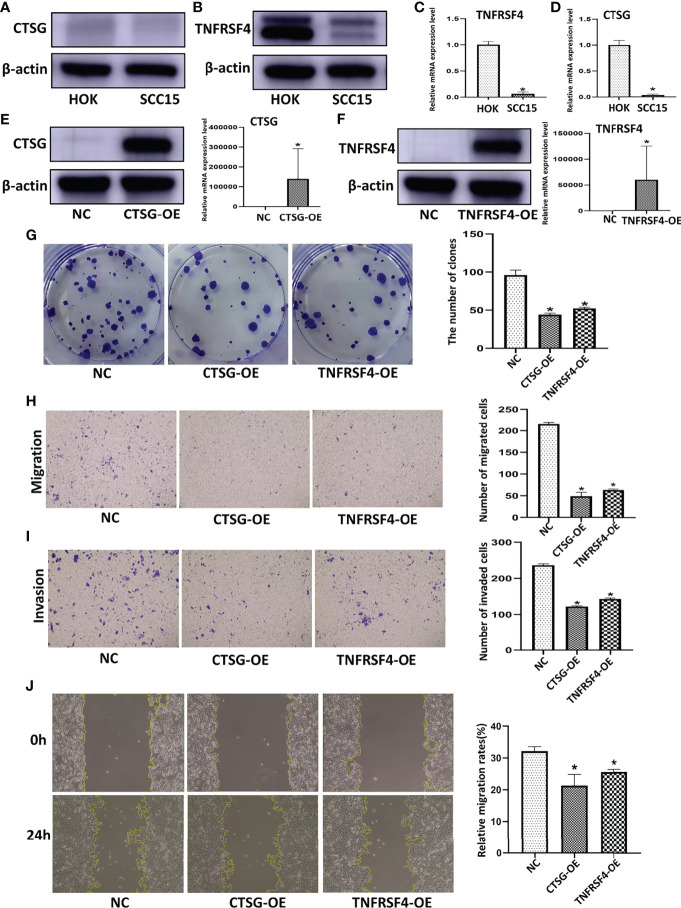
CTSG and TNFRSF4 overexpression using pEXP-RB-Mam-EGFP transfection inhibits OSCC cell line viability and clonogenicity. CTSG **(A)** and TNFRSF4 **(B)** protein expression in OSCC cell lines and normal human oral cavity epithelial cells. CTSG **(C)** and TNFRSF4 **(D)** mRNA expression in OSCC cell lines and normal human oral cavity epithelial cells. CTSG **(E)** and TNFRSF4 **(F)** protein and mRNA expression in OSCC cell lines transfected with pEXP-RB-Mam-EGFP. Colony formation assay of OSCC cell lines treated with specific pEXP-RB-Mam-EGFP and the negative control of CTSG and TNFRSF4 **(G)**. Transwell migration **(H)** and invasion **(I)** assay of OSCC cell lines treated with specific pEXP-RB-Mam-EGFP and the negative control of CTSG and TNFRSF4. Wound healing assays of OSCC cell lines treated with specific pEXP-RB-Mam-EGFP and the negative control of CTSG and TNFRSF4 **(J)**. *P < 0.05

We further performed experimental analysis of genes in prognostic signatures to validate their function in OSCC cell growth and migration. Since CTSG and TNFRSF4 have relatively high coefficient levels and were robust in the previously constructed models, the oncogenic role of these two genes was assessed in further experiments. CTSG and TNFRSF4 protein expression were significantly down-regulated in SCC15 as compared with control HOK cells (**Figures 9A, B**). Similarly, CTSG and TNFRSF4 mRNA expression were significantly lower in SCC15 than HOK cells (**Figures 9C, D**).

To explore the function of CTSG and TNFRSF4 in an OSCC cell line, the pEXP-RB-Mam-EGFP system was used to overexpress CTSG and TNFRSF4 in SCC15 cells. Both CTSG and TNFRSF4 protein and mRNA expression was significantly upregulated by specific pEXP-RB-Mam-EGFP (**Figures 9E, F**). In addition, CTSG and TNFRSF4 pEXP-RB-Mam-EGFP transfection reduced the clonogenicity of SCC15 cells (**Figures 9G**). Furthermore, transwell migration and invasion assays showed that CTSG and TNFRSF4 overexpression significantly reduced SCC15 cell migration and invasion (**Figures 9H, I**). Wound healing assays indicated that SCC15 cell migration steadily decreased following CTSG and TNFRSF4 pEXP-RB-Mam-EGFP transfection *in vitro* (**Figures 9J**). These findings indicated that immune activation correlated with the progression of OSCC malignancy. Anti-CTSG and/or anti-TNFRSF4 medication has been suggested as a potential OSCC treatment strategy.

## Discussion

As one of the most common malignant tumors of the head and neck, OSCC has the characteristics of high heterogeneity and elevated recurrence and metastasis rates (28). While OSCC patient quality of life has improved with significant advancements in surgery, radiotherapy, chemotherapy and multidisciplinary comprehensive sequence therapy, the 5-year survival rate remains low, and this disease is still a serious threat to human health. Smoking and betel nut chewing are the main risk factors for OSCC, however genetic susceptibility, the tumor microenvironment (TME), abnormal gene expression and immune infiltration also correlate with tumorigenesis (29). In recent years, immunotherapy and immune factor-specific targeted therapy are being increasingly used to treat OSCC (30, 31), and existing studies indicate that the immune landscape, such as tumor-infiltrating immune cells (TI), can affect disease progression and are correlated with prognosis and treatment response (32). Therefore, understanding the relationship between OSCC immune cell infiltration and tumor occurrence and development is critical to the design of new methods of diagnosis and treatment. Currently, findings from whole-genome transcriptomics research on cancer imply that immune-related genes can predict cancer patient survival outcomes or responsiveness to certain immunotherapies (33).

The current study used an unsupervised hierarchical clustering approach to create a gene signature that could predict the immune response to OSCC. Using ssGSEA, OSCC patients were categorized into immunity-high and -low groups based on the degree of infiltration of 29 different immune cell types. These results were verified using ESTIMATE and CIBERSORT algorithms, which revealed that there were substantial differences in Stromal, Immune, and ESTIMATE Scores between the high- and low-immune groups. Eighteen immune-related OSCC DEGs that were strongly linked with OS among patients with OSCC were identified in the immunity clusters and ImmPort databases. Among the immune-related genes, hub genes with substantial prognostic significance, including CTSG, CCL22, IGLV1-44, TNFRSF4, and STC2, were identified.

The riskscore of each patient in the TCGA cohort was also determined using the prognostic model, and OSCC patients were divided into high- and low-risk subgroups according to their median riskscore. The five immune-related genes demonstrated reliable and effective predictive abilities in the training set, with patients in the high-risk group having considerably lower OS than those in the low-risk group. Kaplan-Meier survival analysis and the ROC curve were used to verify the five gene prognostic signature in two independent cohorts, the TCGA cohort, and the GSE41613 cohort, after creating the prognostic model. A nomogram was then created to predict OSCC patient outcomes based on the riskscore, age, gender, initial tumor location, and metastatic status of five immune-related gene markers. OSCC survival rates were forecasted for 1-, 3-, and 5-years using the nomogram. The calibration curve demonstrated that this marker could reliably assess the survival rate of OSCC patients.

The immune infiltration state of OSCC was characterized by analyzing differences between the OSCC samples and constructing a prognostic model. Five immune-related genes, CTSG, TNFRSF4, IGLV1-44, STC2, and CCL22, were selected from the immune infiltration cluster as important immune-related prognostic markers. The CTSG gene is located on chromosome 14q11.2 with a span of 2.7kb and consists of five exons and four introns. CTSG is an effective platelet activator and endoprotease that promotes neutrophil effector function by releasing formyl peptide receptor agonists during inflammation (34), induces cell migration, eliminates intracellular pathogens, and causes tissue decomposition in inflammatory areas (35). CTSG is also closely associated with various types of cancer. In human breast cancer MCF-7 cells, CTSG stimulates cell migration and multicellular aggregation using E-cadherin (36). CTSG can also activate pro-MMP-9 to cut and release active transforming growth factor β (TGF-β), MMP-13, and RANKL at the tumor-bone interface of osteolytic lesions induced by breast tumors (37). In addition, CTSG is associated with tumor angiogenesis and metastasis, participating in host defense and neutrophil-related immune responses, and serving as a target of immunotherapy for acute myeloid leukemia (AML) (38). CTSG is also closely related to the survival of several cancer types, including soft tissue sarcoma, muscular invasive bladder cancer, and lymph node-negative breast cancer (39), and is a potential immune-related biomarker for OSCC (40). The current study showed that CTSG could inhibit the proliferation, migration, invasion, and colony formation of SCC15 cells *in vitro*, confirming that CTSG plays a key role in tumorigenesis and progression. TNFRSF4, also known as OX40, is used as a target of immunotherapy for various cancers, including HNSCC, and is associated with a good prognosis (41). Qi et al. found that TNFRSF4 expression differed significantly between Treg subgroups, indicating that it plays an important role in regulating Treg during HNSCC development (42). The current study found that upregulated TNFRSF4 expression correlated with lower SCC15 cell proliferation, migration, invasion, and colony formation. IGLV1-44, a member of the IGLV-subfamily, binds to the non-receptor protein tyrosine kinase TEC family, participates in B cell differentiation, development, proliferation, and apoptosis, and plays an important role in the immune signal transduction process (43). STC2 is a glycoprotein hormone that promotes tumor development and invasion in several human malignancies. Mao et al. showed that STC2 is a potential biomarker for tumor behavior among colorectal cancer patients (44) and a GSEA analysis study reported that STC2 is linked to HNSCC cell growth (45). STC2 is also associated with lymph node metastasis in esophageal cancer patients (46). CCL22 is a chemokine that modulates immunity by increasing Treg contact with dendritic cells in lymph nodes through CCR4 receptor signaling (47, 48). In melanoma, CCL22 boosts Treg recruitment into the TME while inhibiting anticancer immunity (49) and in colorectal adenocarcinomas, CCL22 mRNA expression is considerably higher in tumor tissue than in corresponding normal tissue (50). CCL22 is also associated with Treg and Th1 cells in CRC patients who were exposed to gut microbiota. Wang et al. found that overexpression of CCL22 attracts Th17 cells to induce colon tumorigenesis (51).

The current manuscript describes an immune-related gene model for predicting OSCC outcomes, however, there were some limitations to this study. First, this bioinformatic study was dependent on data from multiple historic datasets. To develop more reliable clinical applications, prospective data from a clinical cohort will be needed to verify the results. While some external experimental validation was conducted, functional research investigations and animal experiments will be necessary to validate the predictive accuracy of the risk model and identify possible immune-related processes.

In summary, hub genes were screened using ssGSEA, and DEGs were discovered using TCGA-OSCC data. Overlapping hub genes identified by ssGSEA and abnormally expressed immune-related genes were used to screen out five immune-related gene prognostic signatures. All five signatures were associated with the prognostic outcomes of OSCC. Biological experiments verified the behavior of these genes in OSCC cell lines. The findings suggest that five identified immune-related gene prognostic signatures may serve as potential immune-related predictive biomarkers for OSCC. An immune-related prognostic signature was developed and confirmed as an independent biomarker with an outstanding ability to predict OSCC outcomes.

## Data Availability Statement

The original contributions presented in the study are included in the article/**Supplementary Material**. Further inquiries can be directed to the corresponding authors.

## Author Contributions

YC and HZ: Contributed to conception, design, data acquisition and interpretation, drafted and critically revised the manuscript. YF, FY and YZ: Contributed to data acquisition and critically revised the manuscript. YG: Contributed to design and critically revised the manuscript. All authors gave their final approval and agree to be accountable for all aspects of the work.

## Funding

This study was supported by the National Natural Science Foundation of China (81800788 and 81773339), Science and Technology Department of Hunan Province, China (2017WK2041 and 2018SK52511), Scientific Research Project of Hunan Provincial Health Commission (202208043514), Natural Science Foundation of Changsha City (kq2202403 and kq2202412), Fund for the Xiangya Clinical Medicine Database of Central South University (2014-ZDYZ-1-16), Education and Teaching Reform Research Project of Central South University (2020jy165-3), Research Project on Postgraduate Education and Teaching Reform of Central South University(2021JGB072), Open Sharing Fund for the Large-scale Instruments and Equipment of Central South University and the Fundamental Research Funds for the Central Universities of Central South University.

## Conflict of Interest

The authors declare that the research was conducted in the absence of any commercial or financial relationships that could be construed as a potential conflict of interest.

## Publisher’s Note

All claims expressed in this article are solely those of the authors and do not necessarily represent those of their affiliated organizations, or those of the publisher, the editors and the reviewers. Any product that may be evaluated in this article, or claim that may be made by its manufacturer, is not guaranteed or endorsed by the publisher.

## Supplementary Material

Supplementary Table 1 The name of 319 immue-related DEGs.

Supplementary Table 2 The details of gene signatures in predicting radiotherapy responses.
